# Differentiation therapy: a promising strategy for cancer treatment

**DOI:** 10.1186/s40880-015-0059-x

**Published:** 2016-01-06

**Authors:** Min Yan, Quentin Liu

**Affiliations:** State Key Laboratory of Oncology in South China, Collaborative Innovation Center for Cancer Medicine, Sun Yat-sen University Cancer Center, 510060 Guangzhou, Guangdong P.R. China; Institute of Cancer Stem Cell, Cancer Center, Dalian Medical University, 116044 Dalian, Liaoning P.R. China

**Keywords:** Differentiation therapy, Cancer treatment, IkB kinase α

## Abstract

Poor differentiation is an important hallmark of cancer cells, and differentiation therapy holds great promise for cancer treatment. The restoration of IkB kinase α (IKKα) leads to the differentiation of nasopharyngeal carcinoma cells with reduced tumorigenicity. The findings by Yan et al. validate the polycomb protein enhancer of zeste homologue 2 (EZH2) as a target for intervention.

Traditional chemotherapy or radiotherapy generally involves killing tumor cells [1, 2]. However, cancer cells may instead be coaxed into becoming normal cells by differentiation therapy, which aims to reactivate endogenous differentiation programs in cancer cells to resume the maturation process and eliminate tumor phenotypes (Fig. 1). Generally, differentiation agents tend to have less toxicity than conventional cancer treatments. A prototype differentiation therapy is all-trans-retinoic acid (ATRA), which induces complete remission in patients with acute promyelocytic leukemia (APL). ATRA induces terminal cell differentiation by disrupting the promyelocytic leukemia/retinoic acid receptor α (PML/RARα) fusion protein that arrests the maturation of myeloid cells at the promyelocytic stage [3]. Subsequently, emerging studies have focused on elucidating the mechanisms of action of differentiation therapy in cancers, particularly in solid tumors. In a paper published on *Nature Communications*, Yan et al. [4] demonstrated that restoring IkB kinase α (IKKα) expression led to cellular differentiation in nasopharyngeal carcinoma (NPC) (Fig. 2).

**Fig. 1:**
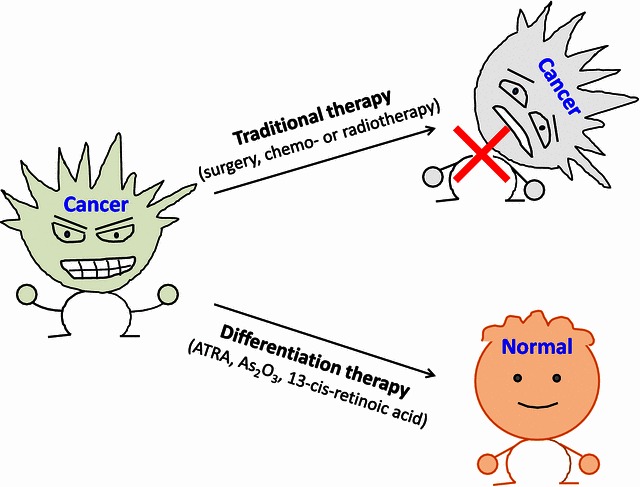
Diagram of differentiation therapy. Compared with traditional cancer treatments, such as surgery, chemotherapy, and radiotherapy that aim to kill tumor cells, differentiation therapy has opened a new door for the treatment of malignant tumors. Differentiation therapy is based on the concept that a neoplasm is a differentiation disorder or a dedifferentiation disease. In response to the induction of differentiation, tumor cells can revert to normal or nearly normal cells, thereby altering their malignant phenotype and ultimately alleviating the tumor burden or curing the malignant disease without damaging normal cells. ATRA, all-trans-retinoic acid

**Fig. 2:**
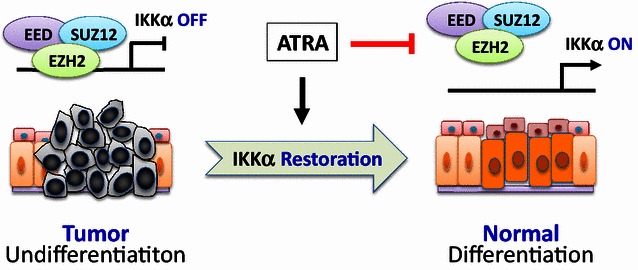
Restoring IkB kinase α (IKKα) promotes nasopharyngeal carcinoma differentiation. In undifferentiated NPC cells, IKKα is epigenetically suppressed by enhancer of zeste homologue 2 (EZH2) (*left*). After ATRA treatment, IKKα is restored, and the tumor cells differentiate towards normal cells (*right*). EED, embryonic ectoderm development; SUZ12, suppressor of zeste 12

NPC is a distinct type of head and neck cancer, and the undifferentiated type (WHO III) is most prevalent (accounting for more than 97% of cases in South China) [5]. The reason for the differentiation block is unclear. This unique characteristic provides an excellent model for exploring the possibility of differentiation therapy in NPC. To identify the key molecules that are essential for NPC cell differentiation, Yan et al. [4] compared three paired RNA libraries from NPC tumors and adjacent non-tumorous tissues, validated their findings by real-time polymerase chain reaction (PCR) and Western blotting assays, and determined that IKKα down-regulation is involved in the undifferentiated status of NPC. This conclusion was also evidenced by the functional analysis, which showed that restoring IKKα in poorly differentiated NPC cell lines (including CNE2, HONE1, and SUNE1) induced differentiation in vitro and decreased tumorigenicity in vivo. IKKα overexpression led to both morphologic and molecular changes in cells that were comparable to well differentiated NP69 and CNE1 cells [4]. Additionally, Yan et al. [4] performed colony formation and nude mouse xenograft assays to reveal the significant suppression of cell proliferation and tumor growth in CNE2-IKKα cells. These findings suggest that IKKα plays a crucial role in NPC differentiation. This function of IKKα is consistent with the result reported by previous studies [6–9] that deleting IKKα resulted in a hyperproliferative and undifferentiated epidermis. Moreover, reintroducing IKKα induces terminal differentiation and inhibits hyperproliferation in IKKα^−/−^ keratinocytes. Although there is clear evidence that decreased IKKα expression is associated with high-grade disease and poor differentiation in human squamous cell carcinoma (SCC) [10, 11], the underlying molecular mechanisms for IKKα repression have not been completely elucidated.

Furthermore, through luciferase reporter and chromatin immunoprecipitation (CHIP) assays, Yan et al. [4] demonstrated that IKKα was epigenetically silenced by an enhancer of zeste homologue 2 (EZH2)-dependent mechanism. Moreover, immunohistochemistry analysis of multiple primary NPC specimens demonstrated that a significant majority of undifferentiated NPC tissues had high EZH2 levels and low IKKα expression, indicating that the phenomenon described for the intensely studied cell line is not isolated.

Importantly, Yan et al. [4] used a classical differentiation reagent, ATRA, to restore IKKα expression and induce the differentiation of poorly differentiated NPC cells. They used a 3-dimentional (3D) cell culture model and showed that after ATRA treatment, the disordered tumor cell mass reorganized into polarized structures compatible with clinical non-cancerous nasopharyngeal specimens. Certainly, it will be interesting to determine the mechanisms by which ATRA reduces the expression of EZH2 and abrogates EZH2-mediated epigenetic repression.

The significance of the study [4] lies not only in its elucidation of IKKα function in NPC differentiation but also in the demonstration of the mechanism for IKKα repression in NPC. Recently, another interesting study reported that restoring the function of the tumor suppressor the adenomatous polyposis coli (APC) causes complete tumor regression with normal differentiation and reestablishes stem cell function in mouse intestinal tumors induced by APC inhibition [12]. Together, these findings enhance our confidence in differentiation therapy and its high translational value in the clinic.

However, many challenges remain for cancer differentiation therapy, particularly in solid tumors. First, the molecular mechanisms responsible for differentiation blocks may vary among different tumor types and patients, even for tumors of equivalent histological class and grade. Second, for the most part, cancer cells cannot completely revert to normal cells with relevant functionality. Third, although many reagents have demonstrated the ability to induce differentiation in preclinical models, few such reagents can be applied in the clinic.

There is evidence that epigenetic regulation plays a crucial role in cell differentiation and embryonic development, as well as in the self-renewal of cancer stem cells [13–15]. Based on the structural and mechanistic complexity of solid tumor differentiation, which is generally regulated by a group of genes, exploring the molecular mechanisms of epigenetic networks may provide new insights for the treatment of solid tumors with differentiation therapy.
